# Evaluating the Effects of 5-Hz Repetitive Transcranial Magnetic Stimulation With and Without Wrist-Ankle Acupuncture on Improving Spasticity and Motor Function in Children With Cerebral Palsy: A Randomized Controlled Trial

**DOI:** 10.3389/fnins.2021.771064

**Published:** 2021-12-15

**Authors:** Jiamin Li, Cen Chen, Shenyu Zhu, Xiulian Niu, Xidan Yu, Jie Ren, Min Shen

**Affiliations:** ^1^Shanghai YangZhi Rehabilitation Hospital (Shanghai Sunshine Rehabilitation Center), School of Medicine, Tongji University, Shanghai, China; ^2^School of Rehabilitation Science, Shanghai University of Traditional Chinese Medicine, Shanghai, China; ^3^Shanghai Taiping Rehabilitation Hospital, Shanghai, China; ^4^Nuffield Department of Women’s & Reproductive Health, University of Oxford, Oxford, United Kingdom

**Keywords:** repetative transcranial magnetic stimulation, wrist-ankle acupuncture, corticospinal tract (CST), motor evoked potential (MEP), spasm

## Abstract

**Objective:** The goal of this study is to explore the effect of wrist-ankle acupuncture combined with 5-Hz repetitive transcranial magnetic stimulation (rTMS) on improving spastic state and motor function of children with spastic cerebral palsy by measuring electrophysiological parameters and behaviors.

**Methods:** Twenty-five children with spastic cerebral palsy were enrolled in a single-blind and randomized controlled trial. The control group received 20 sessions of 5-Hz rTMS over the affected hemisphere with 1,000 pulses. The experimental group was given wrist-ankle acupuncture on the basis of the control group. Gross motor function measure (GMFM-66), muscle tension, and electrophysiological parameters of the two groups were assessed at baseline and after intervention.

**Results:** After treatment, the GMFM-66 scores in the same groups were significantly improved (*p* < 0.001). Besides, the *R*-value of soleus, gastrocnemius, and hamstring muscle decreased (*p* < 0.05), and the results showed a trend of shortening MEP latency, increasing amplitude and duration (*p* < 0.05). Compared to the controlled group, the experimental group displayed more excellent changes in the GMFM-66 scores and motor evoked potential (MEP) latency. The statistical results showed that the increase of GMFM-66 score and the shortening of MEP latency in the experimental group were greater than that in the control group (*p* < 0.05). However, no significant differences were found in the assessment of muscle tension, amplitude, and duration of MEPs between two groups (*p* > 0.05).

**Conclusion:** Wrist-ankle acupuncture combined with 5-Hz rTMS is optimal to improve gross motor function and enhance the conductivity of corticospinal tract in children with cerebral palsy but cannot highlight its clinical superiority in improving spasticity.

**Clinical Trial Registration:** [http://www.chictr.org.cn/index.aspx], identifier [chictr2000039495].

## Introduction

Cerebral palsy (CP) is considered to be a group of persistent central and postural developmental disorders and activity limitation syndrome caused by non-progressive brain injury in fetus or infants, occurring in 1–3 per 1,000 live births (Sellier et al., 2016). Children with spastic CP mainly have motor dysfunction due to different degrees of increased muscle tension and persistent primitive reflex. It is generally believed that the higher central nervous system is damaged, which leads to the obstacle of the central nervous system in the regulation of spinal cord stretch reflex, making the stretch reflex stronger (Kesar et al., 2012). Traditional Chinese medicine believes that the etiology of infantile CP is mostly Yin deficiency, less fluid, and loss of nourishment of muscles, bones, muscles, and joints (Wang Xuefeng, 2005).

Wrist-ankle acupuncture refers to the method of selecting specific needle entry points at the wrist and ankle and using filiform needles to treat diseases by subcutaneous shallow needling along the longitudinal axis of the limb (Zhao et al., 2011). Compared with other acupuncture therapies, it has the characteristics of relative safety, convenient operation, and rapid pain relief and has the function of promoting Qi and blood circulation and mobilizing Wei Qi to regulate the metabolism of human body fluid. The study suggests (Qinghui, 2017) that one of the mechanisms of wrist-ankle acupuncture may be due to the existence of nerve conduction function activities. When the nerve endings are stimulated, it will trigger a series of nerve conduction activities of connecting nerves in the reflex arc and play a complex adjustment role.

Transcranial magnetic stimulation (TMS) is a non-invasive form of brain stimulation that assesses cortical excitability and corticospinal tract conduction through depolarization of corticospinal neurons (Rossini et al., 2015). In many psychiatric and neurological cases, such as depression, the use of 10-Hz frequency, 120% motor threshold, and 3,000 pulses can achieve therapeutic goals (Guse et al., 2010). Besides, it is a valuable supplementary treatment for motor dysfunction, by effectively activating cortical neurons, directly regulating the neurophysiological functions of cortical spinal cord and motor cortex, thus promoting the improvement of motor function (Kamble et al., 2014). According to different parameters, stimulation with frequency ≤ 1 Hz is called low-frequency TMS, whereas stimulation with frequency > 1 Hz is called high-frequency TMS (Kamble et al., 2014). Various studies have shown that high-frequency stimulation increases cortical excitability, whereas low-frequency stimulation suppresses cortical excitability (Marzbani et al., 2018). Children with spastic diplegia and quadriplegia often show bilateral and diffuse brain damage. According to the model of interhemispheric competition inhibition, high-frequency repetitive TMS (rTMS) is often used to increase cortical excitability at the M1 site of the ipsilateral cerebral hemisphere (Valle et al., 2007).

Besides, the security of TMS has been paid more attention (Valle et al., 2007). Krishnan et al. (2015) reviewed the literature on the use of TMS in people under 18 years of age to understand the safety and tolerance ability of non-invasive brain stimulation in children and adolescents, and data from 48 studies in 513 children and adolescents (2.5–17.8 years old) showed that the side effects of non-invasive brain stimulation were generally mild and transient. The side effects after TMS were headache (11.5%) and scalp discomfort (2.5%), convulsions (1.2%), mood changes (1.2%), fatigue (0.9%), tinnitus (0.6%), etc. There are few serious side effects (Krishnan et al., 2015).

In recent years, several studies (Ji et al., 2019) have confirmed that traditional acupuncture combined with rTMS can improve motor function, relieve spasticity and neurotransmitter indexes in children with CP, and promote the improvement of hemodynamics. It can be seen that acupuncture combined with rTMS can further enhance the clinical efficacy compared with a single treatment. Wrist-ankle acupuncture, as one of the means of acupuncture, is a more simplified version compared with traditional acupuncture therapy, and it has the merit of high tolerance for children. This study attempts to use peripheral-central integration model, to further strengthen the clinical effect of children with CP to provide a new way of treatment.

## Materials and Methods

### Participants

Twenty-eight children with spastic CP who received treatment at Shanghai Yangzhi Rehabilitation Hospital between June 2020 and January 2021 were recruited into this trial by an attending physician. However, during the course of treatment and follow-up, there were three cases of rejection and abscission due to personal factors. Finally, 25 cases completed the clinical trial and included the results. The inclusion criteria are as follows: (1) Confirmed diagnosis of spastic CP; (2) aged 3–7 years old; (3) Gross Motor Function Classification System (GMFCS) of CP grading from I to III; and (4) sufficient understanding and cooperation to complete this trial. The exclusion criteria are as follows: (1) The absence of either hemisphere motor evoked potential (MEP); (2) children with organ dysfunction, history of severe epilepsy, cognitive impairment, and other serious diseases; (3) children with CP accompanied by involuntary movements, ataxia, or mixed type; (4) children with artificial pacemaker, cochlear implant, and metal implants; and (5) taken or injected with anti-spasmodic drugs in the past 6 months. The clinical characteristics of participants are shown in Table 1.

**TABLE 1:** Descriptive characteristics of participants.

	Experimental Group	Control Group	*p*-value	t/Z/x^2^
			
	*N* = 14	*N* = 14		
Gender				
Male, N(%)	9(64.3)	10(71.4)	0.686	0.164
Female, N(%)	5(35.7)	4(28.6)		
Age (m)	64.42 ± 21.10	69.28 ± 18.15	0.198	0.653
Height (cm)	116.42 ± 14.37	119.28 ± 12.82	0.638	0.555
Weight (kg)	21.14 ± 6.16	21.92 ± 6.53	0.747	0.323
Classification of cerebral palsy				
Hemiplegia, N(%)	5(35.7)	6(42.9)	0.699	0.150
Diplegia, N(%)	9(64.3)	8(57.1)		
Gross motor function classification system				
Level I, N(%)	8(57.1)	5(35.7)	0.429	1.692
Level II, N(%)	3(21.4)	6(42.9)		
Level III, N(%)	3(21.4)	3(21.4)		

*Continuous variables were presented as mean ±SD.*

### Study Design

**FIGURE 1:**
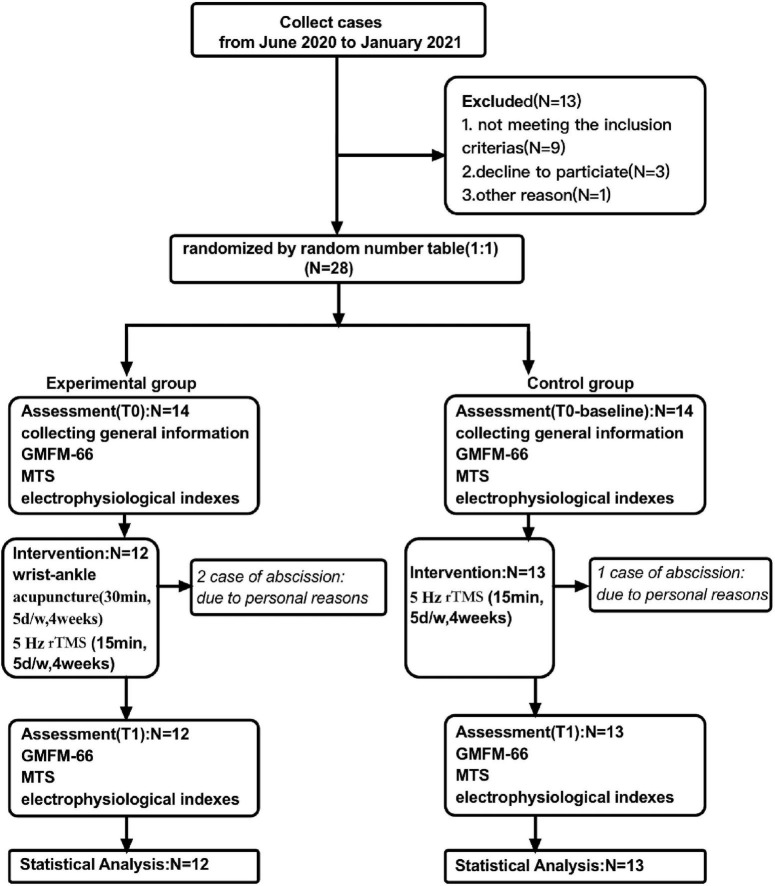
Study design flow chart.

This was a randomized, controlled, single-blind, and parallel-designed prospective clinical trial that was implemented comparing 5-Hz rTMS with and without wrist-ankle acupuncture on motor function and spastic state in children with CP. Ethical approval was obtained by the Medical Ethics Committee of Shanghai Yangzhi Rehabilitation Hospital (YZ2020-066). In addition, the project was registered in Chinese Clinical Trial Registry (no. ChiCTR20000039495). All parents or caregivers have signed informed consent and children verbally agreed to participate in the trial. Participants who met the criteria were randomly assigned (1:1) to two groups (Group A and Group B). The experimental group (Group A) received 20 daily sessions of 5-Hz rTMS over the affected hemisphere with 1,000 pulses. The control group (Group B) was given wrist-ankle acupuncture on the basis of the control group. Assessments were performed at T0 (baseline, 1 week before the intervention onset) and T1 (within 5 days after the intervention). A detailed description of the experimental design is shown in Figure 1.

### Randomization

Randomization was performed by an independent researcher who is not involved in the recruitment procedure, intervention, or evaluation. Participants were randomly assigned into two groups using a random number table in accordance with the 1:1 ratio.

### Sample Size

According to previous study report (Yan-fen, 2015), we selected the scores of GMFM-88 after intervention as a similar reference index. On the basis of the GMFM-88 scores, a margin of 0, an alpha-level of 0.025, a statistical power of 0.90, and a drop rate of 10%, 14 children per group were calculated as the minimum sample size by the formula (Wu et al., 2013; Zhang et al., 2016).

### Blinding

Evaluators and statisticians were all blinded.

### Intervention

The control and experimental groups were conducted in the process of routine rehabilitation, including routine stretch training and strength training. The control used the method of 5-Hz rTMS and the experimental group incorporated wrist-ankle acupuncture treatment in addition to the control. To reduce random error and contingency of the clinical result, wrist-ankle acupuncture was performed first followed by rTMS in the experimental group.

### Repetitive Transcranial Magnetic Stimulation

We utilized TMS devices (made in Denmark with MCF-125 8-shaped coil, model: MagPro × 100) to perform rehabilitation therapy and utilized a neurological diagnosis system (made in Denmark, model: KEYPOINT) to monitor the MEP.

**FIGURE 2:**
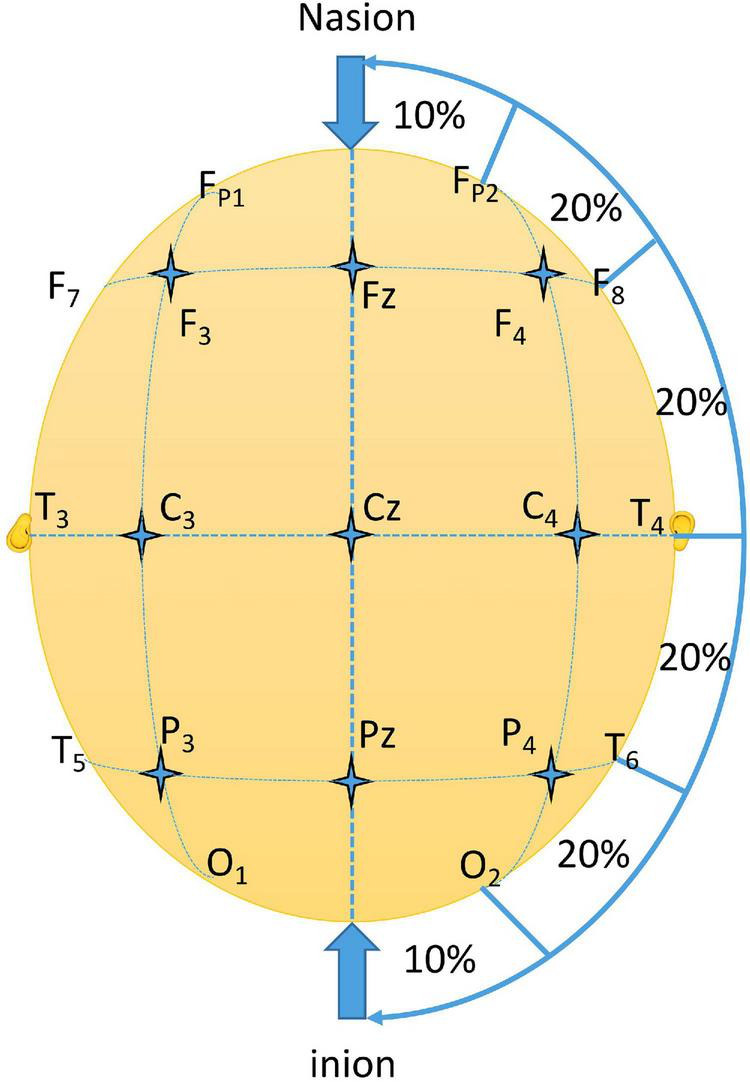
International 10-20 EEG electrode location map.

(1) Resting motor threshold (RMT) measurement: RMT refers to the minimum stimulation that induces target muscle, typically abductor pollicis brevis (APB) muscle to generate MEP over 50 μV in at least five of the 10 single-pulse stimulation (Groppa et al., 2012a; Rossini et al., 2015). Because of individual differences, RMT varies among populations. To monitor the real-time MEP of APB, the patient was placed recumbent or semi-recumbent position and was told to relax. The ground electrode was placed 2-cm proximal to the wrist crease of the forearm. The detection electrode and reference electrode were placed at the muscle belly and the tendon (base of the proximal side of the first phalanx) of APB, respectively. The coil was placed tangent to the surface of the skull and rotated 45° along the sagittal plane. Single-pulse TMS was used to stimulate the corresponding sites of M1 of the head. The output power was first set at 50–60%. If the MEP is not detected after three to five attempts at each site, then the output is increased by 5%. If it reaches 90% of maximum output power and no MEP is induced, then the coil is relocated 0.5 cm front, back, left, and right to the original position, and the same procedure is repeated. Thus, the best site to induce MEP is recorded, and the corresponding RMT is measured and recorded.

(2) Treatment parameters: Subjects were stimulated for 15 min at a frequency of 5-Hz, for a total of 20 sessions (5 days a week, for 4 weeks). The stimulation intensity was set at 90% relative to the observed RMT of the patient, with a stimulus train duration of 5 s and an inter-train interval of 5 s. A total of 40 successive stimulation and 1,000 pulses were performed every time. As the RMT maybe changeable during the whole trial, we would remeasure RMT before daily treatment.

(3) Stimulation site: According to the inter-hemispheric competition inhibition model, a high-frequency stimulation of 5 Hz was performed on the M1 site of the affected hemisphere. If there was no detection of MEP from the ipsilateral M1, the optimal stimulus site is the symmetrical location of the contralateral M1.

(4) Location of M1 (the primary motor cortex): C3 and C4 were located in line with the international 10–20 electroencephalogram (EEG) system on the corresponding sites of the primary motor cortices (Silva et al., 2020). First, the length from nasion to inion was measure and set as 100%. Then, the length between preauricular points was measured and set as 100%. The intersection of the two lines was the point Cz. Four points on the connecting line between the two preauricular points were labeled T3, T4, C3, and C4. T3 and T4 were 10% of the total length between the preauricular points, and the remaining points (including Cz) were all separated by 20% of the total length (Figure 2). Because of anatomical differences, the actual effective stimulation site may differ from the C3 and C4 points. Thus, the exact location of M1 can be determined by moving the coil slightly around the two electrode points to see if there are MEPs.

### Wrist-Ankle Acupuncture Treatment

(1) Acupuncture sites: As stated by Prof. Xinshu Zhang and his summary of acupuncture sites and treatment, the needle insertion points were determined at sections of upper 4, upper 5, lower 1, and lower 4 on the lesion side of the body (Yang et al., 2019).

(2) Method and parameters: Filiform acupuncture needles (Brand: HUATUO, 0.30 × 40 mm) were used. After routine skin disinfection, the site was selected by the criteria. Acupuncture was performed with subcutaneous superficial puncture to avoid feelings of swelling and pain. The insertion was 30° of angle and was performed with single-handed fast insertion and no needle twisting. Then, the needle tail was fixed with medical tape on the skin surface. The patient was told to stay at rest and reduce wrist and ankle movement. The needle insertion lasted for 30 min, once every day, for a total of 20 sessions (5 days a week, for 4 weeks).

### Assessment

#### Gross Motor Function Measure-66

The GMFM-66 consists of 66 items; each item is scored 0–3 points. The final score value is converted by software. On the basis of the original GMFM-88, the GMFM-66 scale was modified and optimized to measure the gross motor function of children with CP in different postures and reflect their motor development level. Compared with GMFM-88, GMFM-66 showed good reliability, validity, and reactivity (Avery et al., 2013).

#### Modified Tardieu Scale—*R*-value

The scale has good reliability and validity in evaluating the degree of spasticity of upper and lower limbs in children with CP. First, move the joint slowly to the “point of sticking” and record the joint angle at this time as R2. Then, move the joint quickly to the “point of sticking” and record the joint angle at this time as R1. Calculate the difference between the two and mark it as *R*(*R* = R2-R1). The improvement of muscle spasm was evaluated by the change of *R*-value before and after treatment (Numanoğlu and Günel, 2012).

#### Latency of Motor Evoked Potentials (MEP LAT)

When the muscle is stimulated, an action potential will be generated, resulting in a negative wave (upward wave). The latency refers to the period from the beginning of the stimulation artifact to the departure of the negative wave from the baseline. It is usually expressed in milliseconds, which represents the excitability of motor cortex and the integrity of corticospinal tract pathway (Kowalski et al., 2019). We recorded the latency five times per subject and calculated the mean of the latency as the measurement of cortical excitability.

#### Amplitude of Motor Evoked Potentials (MEP AMP)

The amplitude refers to the distance between the negative wave peaks and the baseline. It is usually expressed in millivolts. The amplitude of MEP reflects the structural and functional integrity of the corticospinal tract and the excitability of the motor cortex (Van Den Bos et al., 2017).

#### Duration of Motor Evoked Potentials (MEP DUR)

Duration refers to the period from the beginning of the negative wave of action potential deviated from the baseline to returning to the baseline again. Usually, it is expressed in milliseconds. These data can indicate the level of discharge of a single muscle fiber at the same time.

#### Data and Statistical Analysis

Statistical analysis was performed using SPSS version 25.0 (Property of IBM Corp, New York, America) and completed by an independent researcher who is not involved in the recruitment procedure, intervention, or evaluation. If measurement data conforms to normal distribution, the Independent Sample *T*-test and Paired Samples *T*-test are used. If it does not conform to normal distribution, then non-parametric test is used. Pearson chi square test was used for counting data. Wilcoxon rank sum test was used for grading data. Continuous variables were presented as mean ± standard deviation, and *p* < 0.05 was considered statistically significant.

## Results

The subjects of this study were children with spastic CP who received rehabilitation treatment in the outpatient Department of Shanghai Sunshine Rehabilitation Center from June 2020 to January 2021. A total of 28 subjects were included and signed informed consent voluntarily after screening according to diagnosis and to inclusion and exclusion criteria. During the study, two cases were removed from the experimental group, and one case was removed from the control group. They are unable to continue to receive treatment in accordance with the regulations due to personal reasons, such as heavy homework and transfer to other hospitals. Therefore, a total of 25 patients completed the study and were included in the outcome index for statistical analysis, including 12 in the experimental group and 13 in the control group. The clinical characteristics of participants are shown in Table 1.

As can be seen from Table 1, before treatment, there was no statistical significance in gender, age, height, weight, CP classification, GMFCS, and other general data between the two groups (*p* > 0.05), and the data between the two groups were comparable.

As can be seen from Table 2, before treatment, GMFM-66 scores between the two groups were in line with normal distribution, and there was no statistically difference between two groups by independent-samples *t*-test (*p*>0.05), indicating that the data were comparable. At the same time, there was no significant difference in the *R*-values of hamstring muscle, gastrocnemius muscle, and soleus muscle tension between the two groups before treatment (*p* > 0.05). MEP LAT, MEP AMP, and MEP DUR were also compared between the experimental group and the control group before treatment, and there was no significant difference between the two groups (*p* > 0.05). Overall, the data of the two groups were comparable.

**TABLE 2:** Outcome scores at baseline (mean ±SD).

	Experimental Group	Control Group	*p*-value
			
	*n* = 12	*n* = 13	
GMFM-66 MTS (*R*-value)	79.34 ± 11.37	72.46 ± 9.44	0.428
*Hamstring*	16.17 ± 8.87	14.08 ± 4.91	0.956
*Gastrocnemius*	13.33 ± 4.92	10.46 ± 4.62	0.561
*Soleus*	12.50 ± 5.84	12.62 ± 5.49	0.905
MEP LAT	32.49 ± 10.36	29.64 ± 9.48	0.414
MEP AMP	0.07 ± 0.02	0.08 ± 0.02	0.536
MEP DUR	6.03 ± 1.34	6.56 ± 1.32	0.606

As can be seen from Table 3, after treatment, there was significant difference in GMFM-66 scores between the two groups (*p* = 0.026), the increase of score in the experimental group was greater than that in the control group, however there was no significant difference in the *R*-values of hamstring muscle, gastrocnemius muscle, and soleus muscle tension between the two groups (*p*>0.05). Besides, there was significant difference in MEP LAT between experimental group and control group (*p* = 0.026), whereas there was no significance in MEP AMP and MEP DUR (*p*>0.05).

**TABLE 3:** Changes of outcome scores (mean ±SD).

	Experimental Group	Control Group	*p*-value
			
	*n* = 12	*n* = 13	
GMFM-66 MTS (*R*-value)	5.75 ± 2.66	2.81 ± 1.65	0.026
*Hamstring*	8.25 ± 7.98	5.53 ± 4.96	0.435
*Gastrocnemius*	3.41 ± 4.79	1.61 ± 2.25	0.434
*Soleus*	3.66 ± 4.71	3.07 ± 3.81	0.648
MEP LAT	8.65 ± 8.41	2.31 ± 1.67	0.026
MEP AMP	0.07 ± 0.06	0.03 ± 0.04	0.051
MEP DUR	1.23 ± 1.06	1.07 ± 0.76	0.462

**FIGURE 3:**
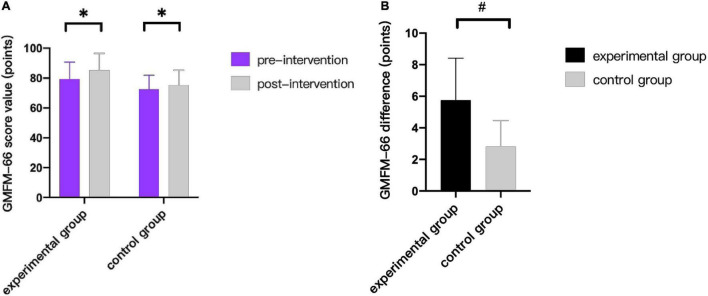
Comparison of GMFM-66 scores. **(A)** Comparison of score value in the same group before and after treatment. **(B)** Comparison of differences between the two groups before and after treatment. * indicates intra-group *p* < 0.05, and # indicates inter-group *p* < 0.05.

**FIGURE 4:**
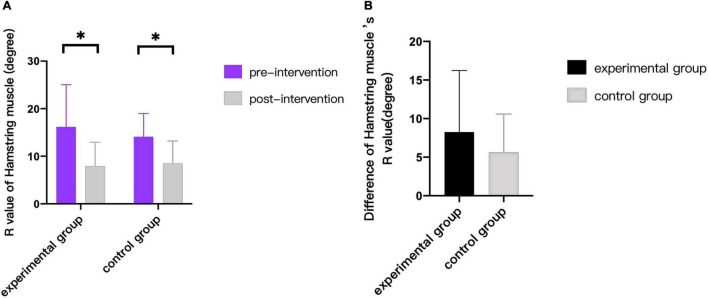
Comparison of R value of hamstring Muscle. * indicates intra-group *p* < 0.05.

**FIGURE 5:**
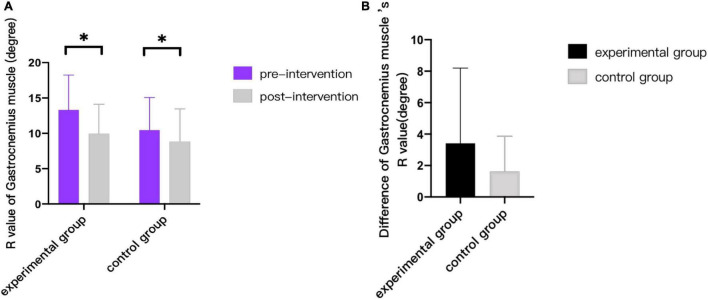
Comparison of R value of gastrocnemius muscle. * indicates intra-group *p* < 0.05.

**FIGURE 6:**
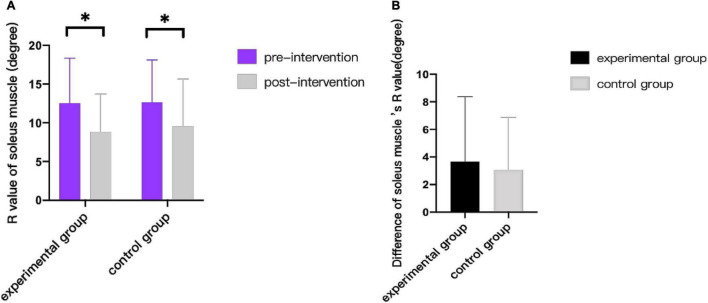
Comparison of R value of soleus muscle. * indicates intra-group *p* < 0.05.

**FIGURE 7:**
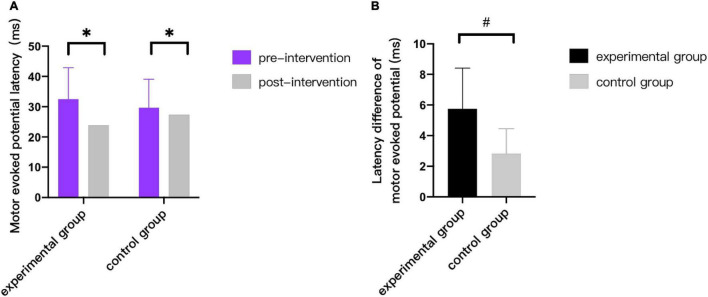
Comparison of latency of motor evoked potentials. * indicates intra-group *p* < 0.05, and # indicates inter-group *p* < 0.05.

**FIGURE 8:**
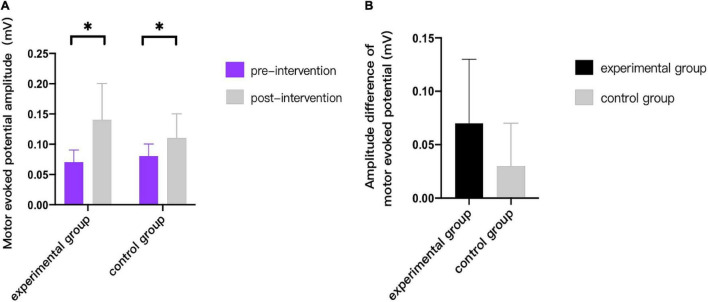
Comparison of amplitude of motor evoked potentials. * indicates intra-group *p* < 0.05.

**FIGURE 9:**
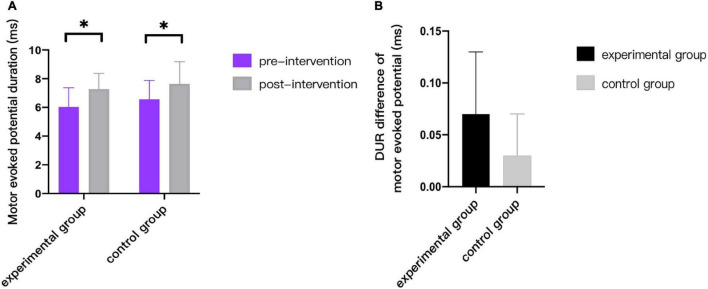
Comparison of duration of motor evoked potentials. * indicates intra-group *p* < 0.05.

As can be seen from Figures 3–9, we can see a change trend of the value. After two courses of treatment, the GMFM-66 scores in the same group were significantly higher than that before, and the difference was statistically significant (*p*_*A*_<0.001, *p*_*B*_<0.001). The *R*-values of hamstring muscle, gastrocnemius muscle, and soleus muscle tension in the same group were lower than that before treatment, indicating significant difference (hamstring: *p*_*A*_ = 0.005, *p_*B*_* = 0.002; gastrocnemius: *p*_*A*_ = 0.027, *p_*B*_* = 0.039; soleus: *p*_*A*_ = 0.011, *p_*B*_* = 0.027). The latency of MEP in the same group was significantly shorter than that before treatment (*p*_*A*_ = 0.002, *p_*B*_* = 0.002). The amplitude of MEP in the same group was significantly higher than that before treatment (*p*_*A*_ = 0.003, *p_*B*_* = 0.016). The duration of MEP in the same group was significantly increased compared with that before treatment (*p*_*A*_ = 0.002, *p*_*B*_<0.001).

## Discussion

This study explored the superiority of wrist-ankle acupuncture combined with 5-Hz rTMS in improving motor function and spasticity in children with CP. From the statistical results, it was found that through the mode of peripheral-central combination, the scores of GMFM-66 and the conductivity of corticospinal tract could be improved. However, it cannot be proved that it has a significant effect on improving spasticity in children with CP in this study. Next, we further discuss the effect and mechanism of wrist-ankle acupuncture combined with 5-Hz rTMS according to previous research reports.

### Effect on Corticospinal Conduction Tract

This study compared the changes of corticospinal tract conductivity between two groups before and after treatment by detecting the electrophysiological indexes such as MEP LAT, MEP AMP, and MEP DUR. The results showed that, after two courses of treatment, both experimental group and control group showed the trend of shortening MEP LAT, expanding MEP AMP and increasing MEP DUR. The difference was statistically significant (*p* < 0.05). At the same time, the decrease of latency in experimental group after treatment was significantly greater than that in control group (*p* < 0.05), but there was no significant statistical difference between two groups on MEP AMP and MEP DUR (*p* > 0.05). The results showed that the conductivity of corticospinal tract was improved in both groups after treatment, but the clinical effect of combined wrist-ankle acupuncture group was better than that of single 5-Hz rTMS group. It can be seen that wrist-ankle acupuncture, as a derivative of traditional acupuncture, plays a certain role and significance in improving neurophysiological function.

The corticospinal tract is the descending nerve conduction tract that controls the voluntary movement of skeletal muscles. The development of pyramidal tracts in bilateral cerebral hemispheres of children with CP is slower than that of normal children; it reflects the disruption of the myelination and axonal integrity of the pyramidal tract during perinatal period, which leads to the dysfunction of motor function (Papadelis et al., 2019). Through single-pulse TMS evaluation, corresponding neurophysiological indexes can be obtained, which can be used as functional biomarkers of brain neuroplasticity and potential therapeutic targets, and further reflect the changes of brain function after non-traumatic brain stimulation intervention (Kirton, 2017). The MEP detected by TMS can be used as a neurobiological marker for the early development of pyramidal tracts in perinatal stroke patients (Kowalski et al., 2019). There is a mechanism that suggests that the shortening of MEP LAT and the widening of amplitude are due to the excitability of α motor neurons and corticospinal tracts (Van Den Bos et al., 2017). The action mechanism of the increase of MEP DUR is explained through research (Brum et al., 2016). It is considered that, during the descending conduction of corticospinal tract, the I α inhibitory interneurons were activated, which further activated the excitatory activity of α motor neurons, and then, MEP DUR increased.

Although the action mechanism of wrist-ankle acupuncture combined with 5-Hz rTMS has not been further studied in this trial, literature studies have found that acupuncture is a bottom-up mode of regulation, specific activation of different brain regions, and there are potential neurochemical mechanisms. By using functional magnetic resonance imaging techniques, researcher found that acupuncture at “Taichong” can trigger increased or decreased signal intensity in several areas of the brain in children with CP (Wu et al., 2008). By exploring the effect of acupuncture on rats with hypoxic-ischemic brain injury, it is found that acupuncture may reduce the degree of neuronal injury after ischemia by inhibiting apoptosis and increasing the expression of glial cell derived neurotrophie factor (GDNF) and brain-derived neurotrophic factor (BDNF), further promote the growth and development, and improve the function of ethology (Zhang et al., 2015). When TMS is applied to the head, it induces action potentials in the cortical axons and spreads across synapses to other neurons, resulting in neuronal activation and spreading of excitation to the adjacent cortical and subcortical regions (Groppa et al., 2012b). At present, there is no specific literature report on the joint action mechanism of wrist-ankle acupuncture combined with 5-Hz rTMS at home and abroad, which can be used as an exploration direction in the future of this study to explore the superiority of the central peripheral closed-loop stimulation mode formed by the combination of the two treatment methods for improving neural electrophysiological function.

### Effect on Spasticity

Through this trial, it is found, that after two courses of intervention, the *R*-value tends to decrease, and muscle tension of the subjects in both groups have been improved to a certain extent (*p*<0.05). It can be concluded that the treatment methods of the two groups have a positive effect on reducing the degree of spasm.

Animal studies have found that (Qi et al., 2014) acupuncture can inhibit the release of inflammatory cells after brain injury, reduce immune response, significantly reduce the muscle tension of spastic CP rats, and increase the activity of spastic limbs. At the same time, the clinical study also found that (Qianfang, 2018) the use of wrist-ankle acupuncture technology in the treatment of children with spastic CP can significantly reduce the muscle tension of the affected side of the upper limb and improve its grasping and visual motor integration ability, and the effect is better than that of traditional acupuncture. It is speculated that the good antispasmodic effect of wrist-ankle acupuncture may be closely related to its analgesic ability.

Pain is common in children with CP (Poirot et al., 2017). Long-term muscle spasticity and skeletal deformity caused by spasm often cause pain (Smith and Field, 2020). The analgesic effect of wrist-ankle acupuncture has been confirmed by many studies. Animal trials have shown that (Liu et al., 2015) wrist-ankle acupuncture can significantly increase the level of nitric oxide in rat tissues and inhibit the production of prostaglandin F2α that causes hyperalgesia and relieves pain. In addition, wrist-ankle acupuncture can activate an endogenous pain regulation mechanism that increases the secretion of β-endorphins and substance P to block the transmission of pain signals.

Besides, Rajak et al. (2019) treated children with spastic CP by rTMS with 10 Hz and 2,500 pulse sequences. The results showed that, after treatment, the degree of upper limb muscle spasm was significantly reduced (Rajak et al., 2019). However, the role of rTMS at different frequencies is also different. Valle et al. (2007) studied the therapeutic effects of low frequency and high frequency on spasticity in children with CP. The results showed that the 5-Hz group had more obvious relief of upper limb spasticity and significantly improved elbow movement than the 1-Hz and sham stimulation groups, and the safety evaluation showed that 1-Hz or 5-Hz stimulation did not cause any side effects in children.

However, there is no significant statistical difference between the experimental group and the control group before and after treatment (*p*>0.05), so the evidence does not support the assumptions that the effect of combined wrist-ankle acupuncture group on improving spasticity in children with CP is better than that in the single 5-Hz rTMS group. The small sample size or low treatment frequency might be the reason. Further experiments are still needed to verify whether this hypothesis is correct.

### Effect on Motor Function

Recent years, acupuncture combined with rTMS were reported to have a more effective impact on motor function when compared to single rTMS (Yan-fen, 2015; Ji et al., 2019. In addition, our statistical results showed that the increase of GMFM-66 scores in the experimental group was greater than that in the control group (*p*<0.05), which revealed the evident superiority of rTMS and wrist-ankle acupuncture combination therapy during motor function recovery.

From the perspective of traditional Chinese medicine, the division of wrist-ankle acupuncture is similar to the 12 skin meridians. The 12 skin meridians are the distribution of the 12 meridians on the body surface. Some theoretical studies refer that the upper 4 areas belong to the hand Yangming large intestine meridian, the upper 5 areas belong to the hand Shaoyang Sanjiao meridian, the lower 1 area belongs to the foot Shaoyin kidney meridian, and the lower 4 areas belong to the Foot Yangming stomach meridian (Qinghui, 2017). The meridians scattered on the skin are adjusted through the selection of needle entry parts at the wrist and ankle, so as to achieve the goal of regulating the viscera, making the Qi and blood sufficient and nourishing the joints.

Several clinical studies investigating the efficacy of wrist-ankle acupuncture in children with spastic CP have reported a large improvement in motor function. These studies (Qianfang, 2018) have suggested that wrist-ankle acupuncture can effectively improve the fine motor ability of both hands of children with spastic CP, and the curative effect is better than traditional acupuncture. Besides, the research results found that (Wenjie, 2019) wrist-ankle acupuncture can significantly improve the pointy foot gait of children with spastic CP and increase the ankle range of motion.

At the same time, rTMS also has a positive effect on the treatment of children with CP. A large number of studies have proved that (Valle et al., 2007; Feng et al., 2013; Rajak et al., 2019) rTMS can improve motor dysfunction in children with CP. Studies showed that (Tan et al., 2014) rTMS can improve cerebral blood flow, improve the metabolic environment of brain cells, delay the cell death cycle, and promote the recovery of brain function. A randomized controlled trial (Feng et al., 2013) discussed the therapeutic effect of rTMS on children with spastic CP. The results showed that, after 1 month of treatment, the sitting ability was significantly improved. After 3 months of treatment, the gross motor functions, such as sitting, kneeling, and climbing, and the fine motor functions, such as range of motion, grasping, and operating objects of limb joints, were significantly improved compared with that in the control group.

This trial found that rTMS combined with wrist-ankle acupuncture has obvious advantages in improving motor function of children with cerebral palsy. In the future, we can also expand the sample size to further explore the persistence of its effect.

## Conclusion

From a clinical point of view, 5-Hz rTMS alone or combined wrist-ankle acupuncture are both of positive significance in improving gross motor function, reducing tension of soleus, gastrocnemius, and hamstring muscles; shortening latency of MEP; increasing amplitude of MEP and prolonging duration of MEP in children with CP; and promoting recovery of motor function by regulating nerve function of corticospinal pathway. However, from a statistical point of view, it can be found that the clinical efficacy of high-frequency rTMS combined with wristankle acupuncture in improving gross motor function and enhancing the conductivity of corticospinal tract in children with CP is better than that of simple high-frequency rTMS, and there is no significant difference in the improvement of spasticity.

## Limitations

Through the results of this study, it could be found that the clinical efficacy of 5-Hz rTMS combined with wrist-ankle acupuncture in improving gross motor function and enhancing the conductivity of corticospinal tract in children with CP was better than that of 5-Hz rTMS alone. In addition, there was no statistical difference in the improvement of spasticity, which may be related to insufficient sample size and low treatment frequency. However, the degree of spasticity was improved according to the change trend of clinical data. Because the influence of COVID-19, recruiting patients during this period was more difficult and time consuming. In the future, it is still necessary to further expand the sample size to increase the reliability of the conclusions. In addition, because the research object is children, there was a problem of compliance in the treatment process. It is necessary to further strengthen the communication with children and parents, so as to obtain better cooperation. At the same time, there is no research report on the joint action mechanism of wrist-ankle acupuncture combined with rTMS, which can be used as an exploration direction in the future to explore the superiority of the central peripheral closed-loop stimulation mode to improve the neurophysiological function. It can also be combined with functional MRI and other imaging methods to explore its mechanism.

## Data Availability Statement

The original contributions presented in the study are included in the article/Supplementary Material, further inquiries can be directed to the corresponding author/s.

## Ethics Statement

The studies involving human participants were reviewed and approved by the Medical Ethics Committee of Shanghai YangZhi Rehabilitation Hospital (YZ2020-066). In addition, the project was registered in Chinese Clinical Trial Registry (No. ChiCTR20000039495). Written informed consent to participate in this study was provided by the participants’ legal guardian/next of kin. Written informed consent was obtained from the individual(s), and minor(s)’ legal guardian/next of kin, for the publication of any potentially identifiable images or data included in this article.

## Author Contributions

JL, CC, SZ, and MS contributed to the design and conception of the study. All authors contributed to the acquisition of data, analysis, and interpretation of the results. JL, CC, and SZ drafted the first version of the manuscript. All authors revised it critically for important intellectual content and approved the final version.

## Conflict of Interest

The authors declare that the research was conducted in the absence of any commercial or financial relationships that could be construed as a potential conflict of interest.

## Publisher’s Note

All claims expressed in this article are solely those of the authors and do not necessarily represent those of their affiliated organizations, or those of the publisher, the editors and the reviewers. Any product that may be evaluated in this article, or claim that may be made by its manufacturer, is not guaranteed or endorsed by the publisher.
